# Development of a Gill Assay Library for Ecological Proteomics of Threespine Sticklebacks (*Gasterosteus aculeatus*)*[Fn FN2]

**DOI:** 10.1074/mcp.RA118.000973

**Published:** 2018-08-09

**Authors:** Johnathon Li, Bryn Levitan, Silvia Gomez-Jimenez, Dietmar Kültz

**Affiliations:** From the ‡Department of Animal Sciences, University of California Davis, Meyer Hall, One Shields Ave., Davis, CA 95616;; §Centro de Investigación en Alimentación y Desarrollo, Carretera a la Victoria Km. 0.6, Apartado, Hermosillo, Sonora, México C.P. 83000;; ¶Coastal Marine Sciences Institute, University of California, Davis

**Keywords:** Label-free quantification, Evolution, Mass Spectrometry, Molecular evolution, SWATH-MS, Targeted mass spectrometry, Data-independent acquisition, Ecological Proteomics

## Abstract

A data-independent acquisition (DIA) assay library for quantitative analyses of proteome dynamics has been developed for gills of threespine sticklebacks (*Gasterosteus aculeatus*). A raw spectral library was generated by data-dependent acquisition (DDA) and annotation of tryptic peptides to MSMS spectra and protein database identifiers. The assay library was constructed from the raw spectral library by removal of low-quality, ambiguous, and low-signal peptides. Only unique proteins represented by at least two peptides are included in the assay library, which consists of 1506 proteins, 5074 peptides, 5104 precursors, and 25,322 transitions. This assay library was used with DIA data to identify biochemical differences in gill proteomes of four populations representing different eco- and morpho-types of threespine sticklebacks. The assay library revealed unique and reproducible proteome signatures. Warm-adapted, low-plated, brackish-water fish from Laguna de la Bocana del Rosario (Mexico) show elevated HSP47, extracellular matrix, and innate immunity proteins whereas several immunoglobulins, interferon-induced proteins, ubiquitins, proteolytic enzymes, and nucleic acid remodeling proteins are reduced. Fully-plated, brackish-water fish from Westchester Lagoon (Alaska) display elevated ion regulation, GTPase signaling, and contractile cytoskeleton proteins, altered abundances of many ribosomal, calcium signaling and immunity proteins, and depleted transcriptional regulators and metabolic enzymes. Low-plated freshwater fish from Lake Solano (California) have elevated inflammasomes and proteolytic proteins whereas several iron containing and ion regulatory proteins are reduced. Gills of fully-plated, marine fish from Bodega Harbor (California) have elevated oxidative metabolism enzymes and reduced transglutaminase 2, collagens, and clathrin heavy chains. These distinct proteome signatures represent targets for testing ecological and evolutionary influences on molecular mechanisms of gill function in threespine sticklebacks. Furthermore, the gill assay library represents a model for other tissues and paves the way for accurate and reproducible network analyses of environmental context-dependent proteome dynamics in complex organisms.

Enormous efforts have been devoted toward development of quantitative proteomics approaches that enable large network-scale analyses of proteome dynamics. As a result, it is now possible to routinely assess systems-scale protein dynamics with superior quantitative accuracy, *e.g.* for quality control of antibody selectivity and specificity (1). An exciting recent development is data-independent acquisition (DIA)^1^ technology, which permits precise and simultaneous quantitation of thousands of proteins (2, 3). This technique is well suited for label-free approaches to be used with virtually any species studied in its natural environment (4, 5). However, application of these technical developments for ecological proteomics is not common and its potential is underutilized. One of the main technical challenges and time-consuming aspects for precise DIA quantitative proteomics is the selection and validation of diagnostically reliable transitions that unambiguously and quantitatively represent unique proteins in diverse samples (6–8). In this study we have addressed this challenge by developing a reliable, standardized, and manually curated DIA assay library for gills of threespine sticklebacks (*Gasterosteus aculeatus*). DIA targeted data extraction has recently been introduced for biomedical applications and shown to be as quantitatively consistent and accurate as selected reaction monitoring (SRM), which is considered the gold standard of quantitative proteomics (3, 7, 8). DIA-based assays combine advantages of selective/multiple reaction monitoring (SRM/MRM) approaches on the one hand and large-scale, high-throughput DDA on the other hand. Like SRM/MRM approaches, DIA methods monitor well-defined transitions representing specific precursors and proteins, although, in DIA the transitions are targeted post-acquisition by using MSMS spectral libraries (9). In addition, DIA approaches are suitable for quantitation of more than a thousand proteins simultaneously, which is why they are sometimes referred to as hyper-reaction monitoring (HRM) (10). The use of the stickleback gill assay library developed in this study was demonstrated by comparing gill proteomes of four stickleback populations adapted to different environments. Quantitative reproducibility of this assay library was verified by analyzing four different population samplings for each of the four habitats.

Threespine sticklebacks are euryhaline fish that represent a long-standing model in behavioral, evolutionary, and ecological physiology research (11). These fish are ancestrally marine, but the species is also represented by many anadromous and land-locked populations that have colonized brackish and freshwater habitats following the retreat of Pleistocene glaciers (12–14). This species is eurythermal but typically restricted to cold-temperate climates throughout coastal areas in the Northern hemisphere (15, 16). However, some exceptional refuge populations have evolved to colonize warm-water habitats (17). These refuge populations provide a unique opportunity for analyzing the genetic and biochemical (*i.e.* proteomic) mechanisms underlying warm-adaptation in vertebrates. Such studies inform us about biochemical coping strategies of vertebrates responding to a changing and stressful climate. Because vertebrates have long generation times, experimental evolution approaches (18) are not feasible. Instead, such studies rely on naturally evolved populations to reveal the mechanisms of environmental adaptation on an evolutionary time scale.

Threespine sticklebacks ideally combine key advantages as a model organism for integrative biology and cross-disciplinary research at the interface of ecophysiology, behavior, evolutionary biology, biochemistry and genetics. They are represented by many populations that have naturally adapted to diverse environments, their complete genome has been sequenced, and their proteome is well annotated (14). A sequenced and well-annotated genome represents a major resource for studying the biology of a species (19). Therefore, much attention has been devoted toward developing and applying whole genome sequencing approaches and strategies for genome, transcriptome, and proteome annotation to an increasing number of species (20). The ability to unambiguously map peptide and protein sequences based on a well-annotated species-specific reference database to unique genomic loci represents a critical prerequisite for proper proteome-wide and unambiguous quantitative analyses. In this study we have used these unique resources and advantages of threespine sticklebacks to develop a strategy for long-term quantitative ecological proteomics of this species. This strategy is based on the generation of validated assay libraries that are tissue-specific and can be used in a highly consistent manner, *i.e.* allowing the quantitation of the same set of proteins based on identical, previously validated precursors and transitions in every sample. To demonstrate its usefulness, the current study has been limited to a single tissue (gill), but the approach can be expanded to include other tissues for integrative studies of environmental effects on whole organism proteomes. The availability of this gill assay library will propel precise, quantitative and network-scale analyses of proteome dynamics in fish experiencing a wide variety of ecological and evolutionary contexts.

## EXPERIMENTAL PROCEDURES

### 

#### 

##### Experimental Design and Statistical Rationale

Population samplings (PSs) were performed at Laguna de la Bocana del Rosario, Mexico, Lake Solano, California, Bodega Harbor, California, and Westchester Lagoon, Alaska. Each PS is represented by six biological replicates (individual fish) from each of the four habitats (24 fish total per PS, see below for population habitat characterization). This experimental design was repeated four times to enable replicated and cumulative population comparisons (PS1, PS2, PS3, PS4 yielding 24 fish per population and 96 fish total). For each fish, gill proteins were extracted and analyzed using procedures outlined below such that each sample analyzed represents an independent biological replicate. For PS1 all 24 samples were processed both by DDA (to create the spectral and assay libraries) and DIA. The remaining 72 samples collected in PS2, PS3, and PS4 were only processed by DIA to assess the robustness of gill proteome population differences detected. Statistical analyses of quantitative DIA results was performed with MSstats, which uses mixed linear models that have been optimized for the quantitative analysis of label-free DIA data in Skyline (21, 22). A power analysis was performed using MSstats and DIA data obtained from PS1 to obtain confidence intervals and determine fold difference thresholds of 2.0 for separate PS comparisons (*n* = 6 per population) and 1.45 for the cumulative comparison (*n* = 24 per population). The false discovery rate (FDR) was set to 1% for protein identification from DDA data. For peak scoring of DIA data, mProphet (23) was used in combination with an equal number of decoy peptides as assay peptides and an FDR of <0.5%. For MSstats (22) statistical analyses of DIA data, the confidence interval threshold was set to >99% and the Benjamini-Hochberg (24) multiple testing-corrected significance threshold was *p* < 0.05.

##### Sample Collection and Preparation

Threespine sticklebacks (*Gasterosteus aculeatus*) were collected from four different habitats using seining and traps as previously described (4). The four habitats were purposely chosen to include populations that represent diverse ecotypes and morphotypes and are adapted to different temperatures and salinities. Thus, the spectral library generated in PS1 is representative of low-plated, warm-adapted, brackish water fish from Laguna de la Bocana del Rosario (Baja California, Mexico), fully-plated, brackish/anadromous fish from Westchester Lagoon (Anchorage, Alaska), low-plated, land-locked freshwater fish from Lake Solano (Winters, California), and fully-plated, resident marine fish from Bodega Harbor (Bodega Bay, California). For brevity these four populations are referred to as LaBoRo, WesLag, LakSol, and BodHar respectively. Their sampling locations, habitat conditions, morphotype, and ecotype are summarized in supplemental Fig. S1. Additional phenotypes for each fish are provided in supplemental Fig. S2. To equally represent both sexes in the spectral library and assay library, three males and three females were included for each population in PS1. Gill dissection, protein extraction, and protein digestion with immobilized trypsin were performed using an optimized sample preparation protocol as described previously (25).

##### Data-dependent Acquisition (DDA)

Tryptic peptides (2 μl, 300 ng/μl) were separated along a 5 to 30% acetonitrile (ACN) gradient over 120 min using a nanoAcquity (Waters, Milford, MA) UPLC and injected into an ImpactHD UHR qTOF mass spectrometer (Bruker Daltonics, Bremen, Germany). The conditions used for DDA were as follows: Peptides were first trapped for 1 min (10 μl/min flow rate) on a trap column (Symmetry, Waters 186003514) and then separated on a 1.7 μm particle size BEH C18 column (250 mm, 75 μm, Waters 186003545) by reversed phase chromatography. The aqueous solvent contained 0.1% formic acid (FA), which was omitted from the organic phase to prevent formation of brown ACN aggregates that would otherwise precipitate at low pH at the pico-emitter tip (New Objective, Woburn, MA, FS360–20-10-d-20). Low concentration tuning mix (Agilent, Santa Clara, CA, G1969–85000) was used daily for mass calibration of the ImpactHD instrument. Batch-processing of samples was controlled with Hystar 4.1 software (Bruker Daltonics). Peak lists were generated with DataAnalysis 4.4 (Bruker Daltonics), imported into PEAKS 8.0 (Bioinformatics Solutions Inc., Waterloo, Canada) and peptide to spectrum matches identified using three search engines (PEAKS 8.0, Mascot 2.2.7, and XTandem Cyclone) followed by unambiguous assignment of peptides to unique proteins using the *G. aculeatus* proteome database downloaded from UniprotKB, version June 27, 2015 (5). The database searched included 27,248 *G. aculeatus* proteins plus the same number of randomly scrambled decoys and 282 common contaminants (human keratins, porcine trypsin, etc.). Trypsin cleavage specificity was C-terminal of either Lys or Arg except when followed by Pro and a maximum of two missed cleavages were allowed. First round search variable PTMs allowed were Cys carbamido-methylation, Met oxidation, Protein N-terminal acetylation and Pro hydroxylation. Second round PEAKS-PTM searches included all 313 variable PTMs contained in the PEAKS database (max. 3 PTMs per peptide). Mass tolerance limits were 0.02 Da for precursors and 20 ppm for fragment ions. PEAKSQ Top3 MS1 quantitative profiling (supplemental Table S1) and Scaffold 4.8 (Proteome Systems, Portland, OR) spectral counting (supplemental Table S2) were performed on PS1 DDA data as previously described (5). Complete data, metadata, and results of the MS1 level surveys performed for PS1 are available at CAMP proteome (AC CAMPDDA00023, https://kueltzlab.ucdavis.edu/CAMP_dda_profiles.cfm?AC=CAMPDDA00023). Data and metadata for these DDA analyses, including the Scaffold file, have been submitted to MassIVE (MSV000081795) and ProteomeXchange (PXD008395) and are publicly accessible under MassIVE project title: Identification and spectral annotation of the threespine stickleback gill proteome.

##### Data-independent Acquisition (DIA)

**Fig. 1.**
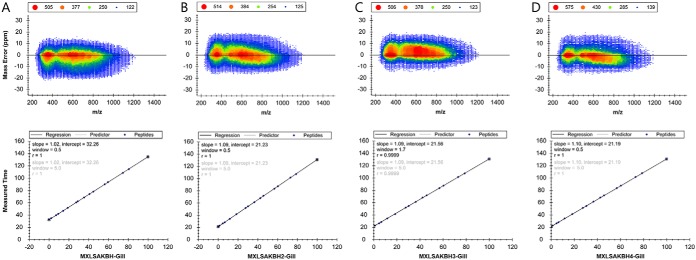
**Mass accuracy and retention time reproducibility for all samples analyzed in this study.** The mass error (ppm) for all transitions present in the final assay library is shown in the top row for PS1 (*A*), PS2 (*B*), PS3 (*C*), and PS4 (*D*). The corresponding retention time reproducibility of the iRT standards is shown in the bottom row. Figures were generated with Skyline 3.6 software (MacCoss Lab., University of Washington).

DIA was performed on all samples collected in all four PSs (96 total biological replicates) using the same instrumentation and separation conditions as for DDA, nanoAcquity (Waters) interfaced online to an ImpactHD UHR qTOF (Bruker Daltonics). Slight variations in retention time (RT) were compensated for by application of internal retention time (iRT) standard calibration in Skyline (Fig. 1). DIA conditions reported previously were used (26) except that the isolation width was set to 10 *m*/*z* (0.5 *m*/*z* overlap) and spectra were collected over 75 isolation width windows ranging from 390 to 1065 *m*/*z* at 37.5 Hz and in 2 s intervals. Except for the acquisition frequency and operating the mass spectrometer in MRM mode, all acquisition parameters were identical to those used for DDA. DIA data from PS1 were used to filter the target list and for selecting iRT standards with Skyline 3.6 (21). Overall system performance and quantitative reproducibility over time was verified weekly by DIA of an aliquot of the same sample (a gill sample from a LakSol stickleback). If necessary, system performance was restored by cleaning the capillary, hexapole cartridge, quadrupole, and collision cell of the mass spectrometer. DIA data and metadata for all four PSs, including all transition, peptide, and document parameters, the final target list, and the *G. aculeatus* gill spectral library have been deposited and are freely accessible at Panorama Public (https://panoramaweb.org/labkey/JoLi-01.url).

##### Spectral Library and DIA Assay Library Construction

**Fig. 2.**
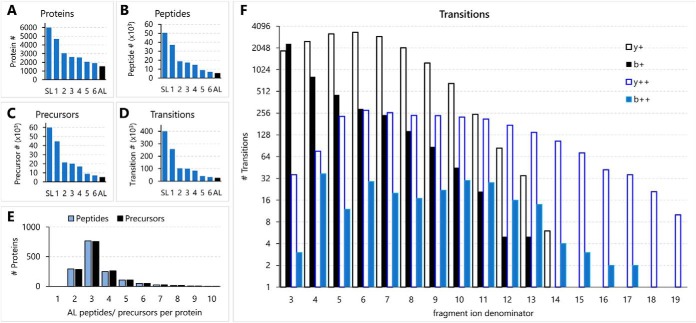
**Composition of the DIA target lists before and after filtration.** The numbers of proteins (*A*), peptides (*B*), precursors (*C*), and transitions (*D*) in the initial (bars labeled SL for Spectral Library) and final (black bars labeled AL for assay library) target lists are depicted. Library filtration steps one to six are explained in the text. *E*, Frequency of proteins represented by two to ten peptides in the final assay library. *F*, Frequency distributions of fragment ion types represented in the final assay library. The data were generated with Skyline 3.6 (MacCoss Lab., University of Washington).

A *G. aculeatus* gill spectral library was generated using the peptide-to-spectrum matches and protein annotation information generated from PS1 DDA data. This information was exported in pepxml and Mascot dat formats and then imported into Skyline 3.6 (21). Skyline was used for spectral library construction and for generating an initial target list, which contained 400,412 transitions, 60,132 precursors, 50,501 peptides, and 5998 proteins (Fig. 2*A*–2*D*). Transitions for the initial target list were automatically chosen from library spectra using the Skyline transition settings dialogue based on the following criteria: ion 3 to last ion −1; exclusion of the precursor isolation window (*m*/*z* width = 10); charge 1, 2; precursor charge 1–4; mass accuracy threshold within ±0.035 m/z of the expected mass. This initial target list was filtered sequentially in seven steps using Skyline. The impact of each filtering step on the number of transitions, precursors, peptides, and proteins lost from the assay library is shown in Fig. 2*A*–2*D*. Filter step one consisted of exclusion of PTMs other than Met oxidation and Cys carbamido-methylation. During filter step two all repeated peptides were excluded and peptide uniqueness by proteins was enforced. Filter step three excluded all precursors with less than four transitions and filter step four excluded all peptides with any missed cleavages. The remaining three filter steps were performed using a sample training set, which consisted of all 24 PS1 samples (six from each of the four populations). All training samples were subjected to iRT calibration and mProphet (23) peak detection using Skyline as follows: First, iRT calibration was performed based on 16 internal standard peptides (Fig. 1*A*, for iRT standard peptides see Panorama Public, https://panoramaweb.org/labkey/JoLi-01.url). The Skyline mProphet (23) module was then used for optimizing peak detection and scoring after generating randomly scrambled decoy peptides equal in number to assay peptides. The q value threshold for mProphet peak detection was set at 0.005 (FDR < 0.5%). After mProphet peak detection the iRT calibration was repeated to correctly reflect the RT of optimized peaks. After iRT calibration and mProphet peak scoring for the 24 PS1 samples, it was possible to apply three additional filter steps that considered the quality of peaks for each peptide in the assay library. The advanced refine feature of Skyline was used in step five to exclude all peptides with dotp values <0.8 in all 24 samples and in step six to exclude all proteins that were represented by less than two remaining peptides. The final filter step (seven) consisted of curation of the assay library by manual inspection of the peak shapes for all peptides in the 24 PS1 samples. This step was performed independently by two investigators and resulted in removal of remaining low intensity peptides and large interferences. The rules followed for manual curation were as follows: (1) If an interference was greater than the actual peak at proper RT then the corresponding transition was removed; (2) If a precursor peak is not clearly discernable in at least half the samples of one population within 3 min of the predicted RT then the precursor was removed; (3) If precursor peak widths differ by more than 2-fold for half of the samples of the same population from the other half, then the precursor was removed; (4) If more than one transition of the five nominal transitions shows an interference that exceeds the intensity of the actual transition peak at the predicted RT then the corresponding precursor was removed. The resulting final target list contains 25,322 transitions, 5104 precursors, 5074 peptides, and 1506 proteins (Fig. 2*A*–2*D*). The complete assay library including all relevant metadata is available at Panorama Public (https://panoramaweb.org/labkey/JoLi-01.url). This assay represents a tier two assay (27).

##### Statistical Analyses and Visual Data Representation

**Fig. 3.**
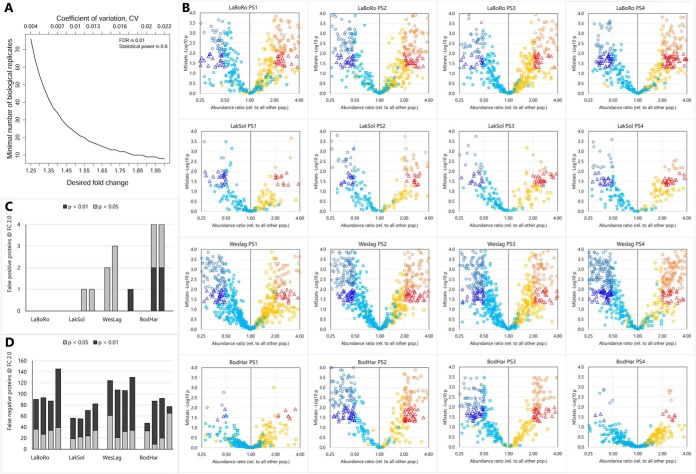
*A*, Fold change (FC) and coefficient of variation (CV) depending on number of biological replicates at a statistical power of 0.8 and false discovery rate (FDR) of 1%. *B*, Volcano plots for each population sampling (PS) and each population (Laguna de la Bocana del Rosario = LaBoRo, Lake Solano = LakSol, Westchester Lagoon = WesLag, Bodega Harbor = BodHar). For clarity, only proteins that are significant in at least one PS and fall within FC −4 (0.25) to +4 and -log 10 p between 0 and 4 are plotted. For complete coverage refer to supplemental Table S4. Blue circles represent proteins that are significantly reduced in all PSs at *p* < 0.01 and FC ≥ 2, blue triangles represent proteins that are significantly reduced at *p* < 0.05 and FC ≥ 2 but not at *p* < 0.01, blue squares represent proteins that are not significantly reduced for the corresponding PS. Orange circles represent significantly elevated proteins at *p* < 0.01 and FC ≥ 2, red triangles represent significantly elevated proteins at *p* < 0.05 and FC ≥ 2 but not at *p* < 0.01, yellow squares represent proteins that are not significantly elevated for the corresponding PS. *C*, Number of false positive proteins at *p* < 0.05 and *p* < 0.01 (cumulative bars) for PS1 to PS4 (from left to right) and each population. Missing bars indicate zero false positives. False positives are expressed as the number of proteins exceeding the significance threshold in the opposite FC direction for the PS depicted compared with any of the other three PSs (*e.g.* FC = 2.0 in PS1 but 0.5 in PS2). *D*, Number of false negative proteins at *p* < 0.05 and *p* < 0.01 (cumulative bars) for PS1 to PS4 (from left to right) and each population. False negatives are expressed as the number of proteins exceeding the FC threshold of 2.0 but not the statistical significance threshold.

Quantitative DIA data were normalized at the MS2 level by equalizing medians in each sample followed by comparison of protein quantities in each population against all other populations with the MSstats (22) module integrated in Skyline using the following parameters: confidence interval > 99% (FDR < 1%), scope = protein, summary method = Tukey's median polish, use zero for missing peaks, and q value < 0.005. The q value represents the FDR associated with the mProphet peak scoring model applied to the DIA data, *i.e.* mProphet FDR < 0.5%. The manual curation was the final step (step seven) after mProphet peak scoring during library assay generation. This final step was only performed for PS1 samples, which were used as training samples for steps five to seven of assay library construction (see previous paragraph). Once the assay library had been established using PS1 samples no manual curation was performed for subsequent DIA data sets (PS2, PS3, and PS4). Rather, the same assay library, parameters, and thresholds as used for PS1 were applied in conjunction with iRT calibration and mProphet peak scoring to quantify transition peaks for all subsequent DIA data sets (PS2, PS3, and PS4). Reproducibility of proteins identified as significantly different in abundance was assessed using two common multiple testing-corrected *p* value thresholds (0.01 and 0.05). This analysis demonstrates that a multiple testing-corrected *p* value threshold of 0.05 represents the best compromise for balancing false positives with false negatives (Fig. 3). DIA data exported from Skyline were used for generating heat maps with Genesis 1.8.1 (28). The Venny 2.1 tool was used for visual representation of data as Venn diagrams (29).

##### Pathway and Gene Ontology Analyses

The UniprotKB ID mapping tool was used to annotate stickleback gill proteins with their corresponding KEGG (Kyoto Encyclopedia of Genes and Genomes https://www.kegg.jp/) orthology (KO) identifiers. KO identifiers were assigned to 1307 of the 1506 proteins contained in the final assay library (supplemental Table S3). Sets of proteins that were significantly enriched/depleted in each of the four populations were then subjected to pathway analysis using the KEGG Mapper tool (https://www.genome.jp/kegg/tool/map_pathway1.html). This approach identified corresponding KEGG pathway maps that contain proteins for each of the population-specific enriched/depleted sets. Pathway maps containing 5 proteins or more per set were considered most informative. PANTHER (Protein Annotation Through Evolutionary Relationships) was used for gene ontology (GO) and pathway enrichment analysis as previously described (4, 5, 30). A hidden Markov model (HMM) based gene ontology PANTHER library for the entire threespine stickleback proteome constructed on January 25, 2015 was used for this purpose (5). This library contains the PANTHER IDs for all but 12 of the 1506 gill assay library proteins (supplemental Table S3). Stickleback PANTHER IDs of population-enriched/depleted protein sets were searched against the complete assay library of 1506 proteins to identify significantly enriched and depleted GO categories and pathways with the PANTHER enrichment test (Release date: April 13, 2018). A multiple-testing corrected PANTHER enrichment test *p* < 0.05 was considered statistically significant for enrichment analyses.

## RESULTS

### 

#### 

##### Gill Proteome Identification and Spectra Annotation

Unbiased DDA proteome profiling of all samples collected in PS1 followed by PEAKS 8.0 (Bioinformatics solutions), Mascot 2.2.7 (Matrix Science Inc.), and X!Tandem Cyclone (the GPM) database searching unambiguously identified 2101 unique proteins that are represented by 17,568 unmodified peptides (MassIVE AC MSV000081795). In addition, label-free quantitation of protein abundances using the MS1-Top3 approach with PEAKSQ (determination of chromatographic peak area for the three most abundant, unique MS1 peptides) yielded many candidate proteins that are differently abundant in the four populations (supplemental Table S1, CAMP proteome AC CAMPDDA00023). After consolidating database search results from multiple search engines (PEAKS, Mascot, and X!Tandem) in Scaffold 4.8 (Proteome Systems Inc., Portland, OR) the complete dataset contained 1652 proteins represented by at least two unique peptides at 1% protein FDR and 0.1% peptide FDR (supplemental Table S2, CAMP proteome AC CAMPDDA00023). The Scaffold file also contains semi-quantitative spectral count analyses that suggest candidate proteins whose abundances are population-specific (MassIVE AC MSV000081795, CAMP proteome AC CAMPDDA00023). The PEAKS Top3 and Scaffold spectral counting results obtained from PS1 indicate two broad trends: First, gill proteomes differ markedly between the four populations. Second, the gill proteomes of the LaBoRo and WesLag populations are most unique compared with the other populations. For the purpose of this study the DDA data were used for assay library generation and not for systematic comparison of Top3 and spectral counting approaches with assay library quantitation, which exceeds the scope of this article.

##### Generation of the Spectral Library and cDIA Assay Library

Search results for DDA data of all 24 PS1 samples were obtained by multiple search engines, consolidated with PEAKS Studio, and used for MSMS spectral library construction. A highly diverse set of samples was purposely collected from different populations, morphotypes (fully- and low-plated), and ecotypes (resident marine, resident freshwater, and brackish water) of *G. aculeatus* adapted to different temperature, salinity, and latitude (supplemental Fig. S1). This sample diversity supports a wide representation of pertinent *G. aculeatus* proteins in the spectral library and empowers systematic future analyses of differentially abundant proteins in diverse populations.

The initial spectral library contains a much greater number of transitions, peptides, precursors, and proteins than what is included in the final assay library (Fig. 2*A*–2*D*). This large difference is because of redundancy, ambiguity, and low-quality targets that are removed during target list filtering. DIA was performed on all 24 PS1 samples to assess the two broad trends in population-dependent proteome dynamics outlined above more precisely. The resulting PS1 DIA dataset was used as a training sample set for target list filtering to generate the final assay library. The numbers of peptides and precursors representing each protein included in the final, filtered assay library are depicted in Fig. 2*E*. Most proteins are represented by three precursors/peptides and the average number of precursors/peptides per protein in this assay library is 3.9. Almost all precursors in the assay library (4881) are represented by the maximum number of five transitions with the remainder being represented by four transitions. Almost all peptides in the assay library are represented by a single charge state and only 15 peptides are represented by two precursors. The most common precursor charge in the assay library is 2+ (4118 precursors) followed by 3+ (870 precursors), 4+ (66 precursors), and 1+ (47 precursors). The most frequent transition ions in the assay library are y4+, y5+, y6+, y7+, y8+, and b3+ (Fig. 2*F*). The complete optimized *G. aculeatus* gill assay library can be downloaded from Panorama Public (https://panoramaweb.org/labkey/JoLi-01.url) and CAMP proteome (https://kueltzlab.ucdavis.edu/CAMP_dia_exp.cfm?ac=CAMPDIA00001).

##### Reproducibility of Population-specific Proteome Signatures

Power analysis performed with MSstats yielded a fold change (FC) threshold of 2.0 when each PS was analyzed separately (*n* = six per population) and 1.45 when all four PSs where combined (*n* = 24 per population, Fig. 3*A*). The number of false positives and false negatives was estimated at the FC threshold of 2.0 at two different multiple-testing corrected alpha levels (*p* < 0.05 and *p* < 0.01). Proteins that exceed this FC of 2.0 in any of the four separate PSs (PS1, PS2, PS3, PS4) were graphed on volcano plots for each population and each PS (Fig. 3*B*). The false positive rate was estimated as the number of proteins from this subset that display the opposite direction of regulation in any of the four PS at significance thresholds of *p* < 0.05 and *p* < 0.01. The false negative rate was estimated as the number of proteins from this subset that exceed the FC threshold of 2.0 in the correct direction but do not reach the significance threshold (*p* < 0.05 and *p* < 0.01) in a particular PS. The number of false positive proteins defined in this way is very low. More importantly, the increase in the number of such false positive proteins is very small when the significance threshold is changed from *p* < 0.01 to *p* < 0.05 (Fig. 3*C*). In contrast, the number of false negative proteins is much higher, and it increases greatly when the significance threshold is changed from *p* < 0.05 to *p* < 0.01 (Fig. 3*D*). Therefore, and because correction for multiple testing already treats *p* values conservatively, a significance threshold of *p* < 0.05 was applied to quantitative population comparisons performed with the DIA assay library.

The four repeated PSs enabled validation of proteins with significantly and at least 2-fold different abundances in a single population relative to the other populations. Data from all four PSs had a mass error less than 10 ppm for a great majority and less than 20 ppm for all MS2 spectra collected from all samples (Fig. 1). Retention time reproducibility was also very high for all samples in all four PSs as illustrated by the elution profiles of iRT standards (Fig. 1). General trends observed in PS1 and the magnitude of differences between populations as illustrated by volcano plots were reproducible in the other three PSs (Fig. 3). The only exception is the BodHar population for which much fewer abundance differences have been identified in PS1 and PS4 than in PS2 and PS3 (Fig. 3). Volcano plots illustrate that some differences between PSs regarding the sets of proteins identified as significantly differentially abundant in each of the populations are to be expected. They show that many of these proteins cluster near the 2-fold threshold, which is exceeded in some but not all PSs (Fig. 3).

Almost all proteins that were significantly altered in a population in one PS were also altered in the same FC direction in the other PSs (Fig. 3*B*, supplemental Table S4). Even though statistical significance (*n* = six per population, *p* < 0.05, FC ≥ 2.0) was not reached for many of these proteins in all four PSs, highly reproducible proteome signatures are evident for all populations. The LaBoRo proteome signature consists of 33 proteins that are significantly elevated and 23 proteins that are significantly reduced in all four PSs (supplemental Table S4). The LakSol proteome signature consists of six proteins that are significantly elevated and two proteins that are significantly reduced in all four PSs (supplemental Table S4). The WesLag proteome signature consists of 30 proteins that are significantly elevated and 29 proteins that are significantly reduced in all four PSs (supplemental Table S4). The BodHar proteome signature consists of one protein (glutamine synthetase) that is significantly elevated and one protein (transglutaminase 2) that is significantly reduced in all four PSs (supplemental Table S4). In summary, even with a relatively low number of biological replicates (six fish per population) the four PSs illustrate that robust population-specific proteome signatures consisting of many reproducibly statistically significant proteins can be obtained with the DIA assay library approach. In addition, these proteome signatures illustrate that LaBoRo and WesLag gill proteomes differ most from those of the other populations whereas the BodHar proteome signature is the least unique.

##### Biological Relevance of Population-specific Proteome Signatures

**Fig. 4.**
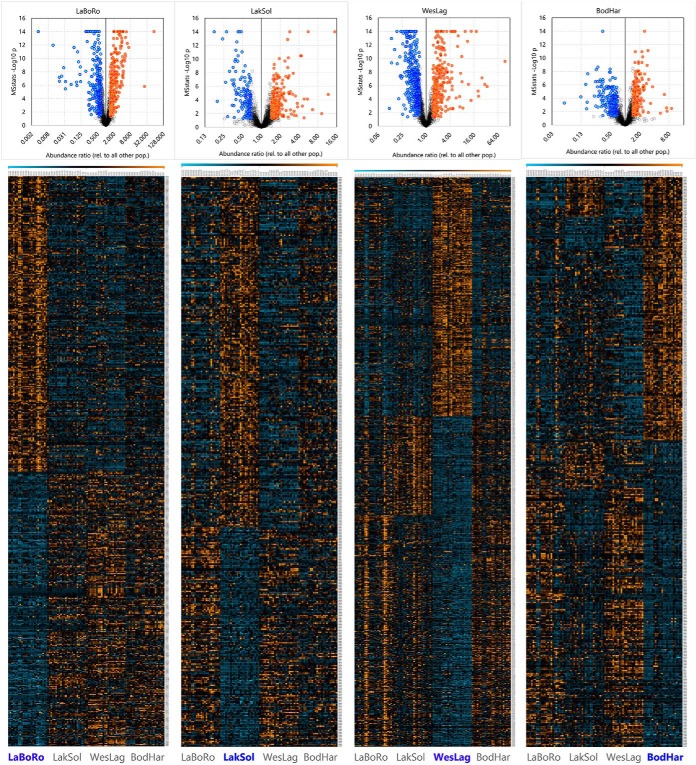
**Proteins from threespine stickleback gills that are significantly elevated and reduced in each population relative to the other populations.** Data from all population samplings were combined for this analysis. The top row shows the volcano plots with significant proteins depicted in blue (reduced) and orange (elevated). If -Log10 p values exceed 14 then they are depicted as 14. Underneath the volcano plots are the corresponding heat maps sorted by first elevated and then reduced proteins. Each heat map depicts data for 96 biological replicates from left to right as follows: 24 LaBoRo, 24 LakSol, 24 WesLag, 24 BodHar. The color scale of the heat map ranges from −3 (3-fold reduction, blue) to +3 (3-fold increase, orange). Note that the number of significant proteins is different for each population and that heat maps are stretched to fit the panel. These data are also available in numerical form in supplemental Table S5.

Samples from all four PSs were pooled into a single comparison to identify more comprehensive proteome signatures that are specific for each of the four populations. Pooling samples reduces the FC threshold from 2.0 to 1.45 at the same statistical power (Fig. 3*A*). Analysis of the combined set of samples including all four PSs with MSstats yields 258 proteins that are significantly elevated and 242 proteins that are significantly reduced in LaBoRo fish (Fig. 4, supplemental Table S5). The WesLag population is characterized by 293 significantly elevated and 406 significantly reduced proteins (Fig. 4, supplemental Table S5). The other two populations have fewer significantly elevated (231 for LakSol and 178 for BodHar) and reduced (145 for LakSol and 207 for BodHar) proteins (Fig. 4, supplemental Table S5). The corresponding volcano plots verify that there is no bias in overall protein abundance for any of the populations. They also show that gill proteomes of the LaBoRo and WesLag populations are more unique (*i.e.* proteins have larger FC and higher statistical significance) than those of LakSol and BodHar populations (Fig. 4).

**Fig. 5.**
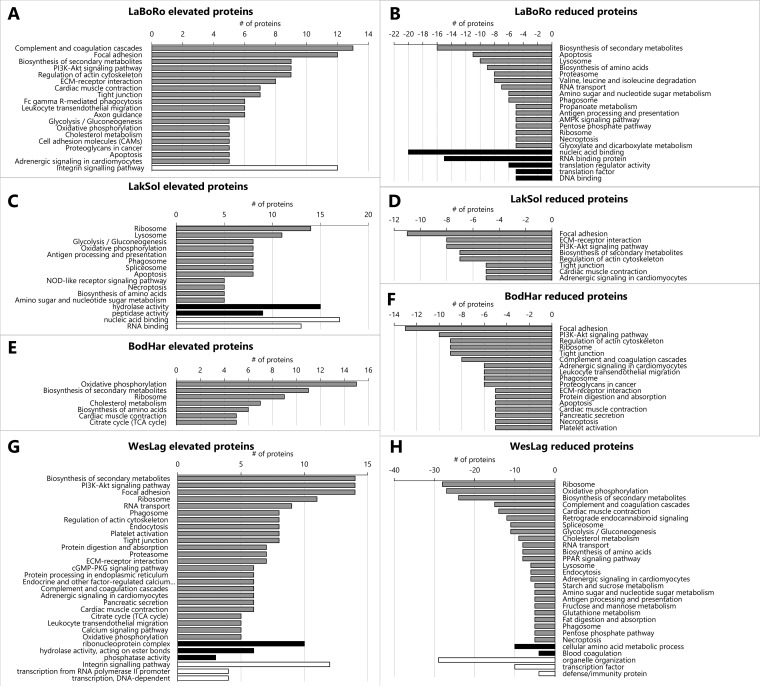
**Numbers of significantly elevated (left panels) and reduced (right panels) proteins that cluster in KEGG pathways (minimum = 5, gray bars) and are significantly enriched (black bars) or depleted (white bars) in PANTHER pathways and gene ontology (GO) categories.** Data are shown for the Laguna de la Bocana del Rosario (*A*, *B*), Lake Solano (*C*, *D*), Bodega Harbor (*E*, *F*), and Westchester Lagoon (*G*, *H*) populations.

**Fig. 6.**
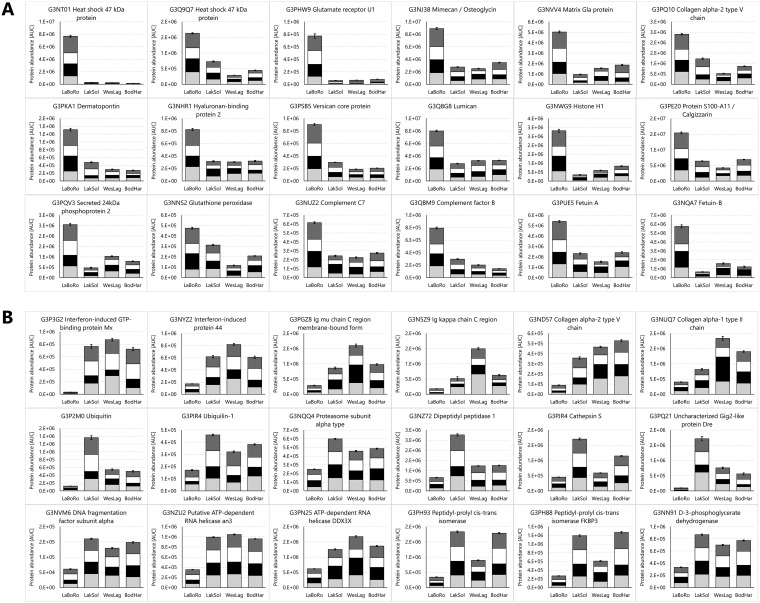
**Proteins from threespine stickleback gills with the greatest and most significant abundance differences in the Laguna de la Bocana del Rosario (LaBoRo) population compared with the other populations.** Data for significantly elevated (*A*) and reduced (*B*) proteins represent the sum of the means of all four population samplings (PSs). Error bars represent the S.E. of the cumulative dataset from all four PSs (*n* = 24 per population). For each protein the reproducibility of the overall trend can be assessed by inspecting the contributions of individual PSs, which are indicated by different black and white shading intensity (PS1 to PS4 from bottom to top).

KEGG pathway and PANTHER GO term and pathway enrichment analyses revealed certain pathways and biological functions that are associated with population-specific proteome signatures. Many KEGG pathways and PANTHER GO terms and pathways are represented by the 1506 proteins included in the assay library (supplemental Tables S5 and S6). PANTHER enrichment analyses using the population-specific sets of significantly elevated and reduced proteins against the complete assay library set of 1506 proteins reveals that the integrin signaling pathway is depleted (*p* = 0.0301) among elevated LaBoRo proteins whereas nucleic acid binding (*p* = 0.00734), RNA binding (*p* = 0.00357), DNA binding (*p* = 0.0349), translation regulator activity (*p* = 0.00364), and translation factors (*p* = 0.001) are enriched among reduced LaBoRo proteins (Fig. 5*A*, 5*B*). Moreover, KEGG pathways represented by at least five proteins elevated in the LaBoRo population indicate a remodeling of extracellular matrix, cell adhesion, plasma-membrane anchored cytoskeleton, and the complement system (Fig. 5*A*). Different apoptosis proteins are represented in KEGG pathways associated with elevated and depleted LaBoRo protein sets suggesting that apoptosis is also remodeled in this population. Furthermore, proteasome, lysosome, ribosome, amino acid synthesis, RNA processing, antigen processing and presentation, and necroptosis pathways are represented by sets of at least five proteins that are reduced in the LaBoRo population indicating that protein turnover and adaptive immunity are reduced (Fig. 5*B*). Pathways that are uniquely regulated in LaBoRo fish are reflected in the most highly and significantly elevated and reduced proteins. Two HSP47 isoforms, many extracellular matrix proteins, complement proteins, and cell adhesion regulatory proteins are most highly and significantly elevated whereas several interferon-induced proteins, immunoglobulins, ubiquitins, proteases, DNA/RNA processing proteins, and peptidyl prolyl cis/trans isomerases are most reduced in LaBoRo fish (Fig. 6).

**Fig. 7.**
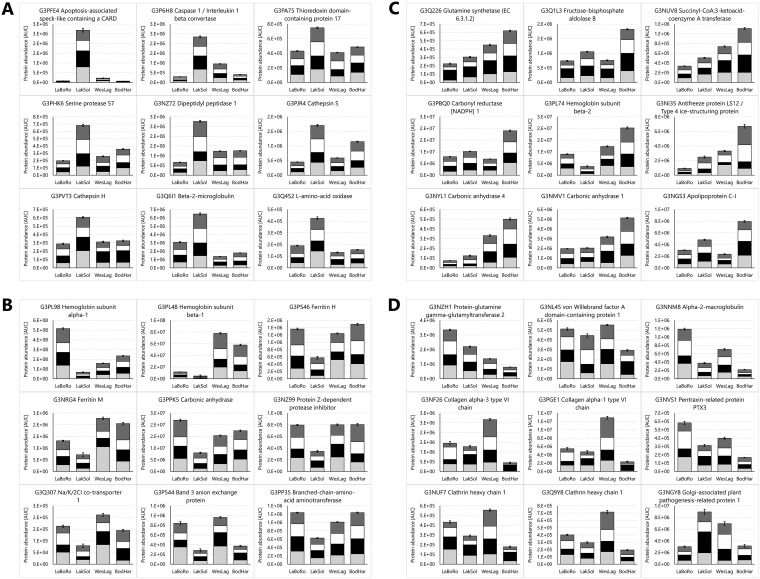
**Proteins from threespine stickleback gills with the greatest and most significant abundance differences in the Lake Solano (LakSol, *A* and *B*) and Bodega Harbor (BodHar, *C* and *D*) populations compared with the other populations.** Data for significantly elevated (*A, C*) and reduced (*B, D*) proteins represent the sum of the means of all four population samplings (PS). Error bars represent the S.E. of the cumulative dataset from all four PSs (*n* = 24 per population). For each protein the reproducibility of the overall trend can be assessed by inspecting the contributions of individual PSs, which are indicated by different black and white shading intensity (PS1 to PS4 from bottom to top).

PANTHER enrichment analyses for the set of elevated LakSol proteins shows that hydrolase (*p* = 0.00129) and peptidase (*p* = 0.00257) activities are enriched whereas nucleic acid (*p* = 0.00187) and RNA (*p* = 0.00136) binding proteins are depleted (Fig. 5*C*). KEGG pathways represented by at least five proteins that are elevated in the LakSol population indicate proteostasis and immunity related processes (ribosome, lysosome, phagosome, antigen processing/presentation, apoptosis, necroptosis) are enriched (Fig. 5*C*). KEGG pathways represented by reduced LakSol proteins include focal adhesion, ECM-receptor interaction, tight junctions, PI3K-Akt signaling, actin cytoskeleton regulation, and oxidative metabolism (Fig. 5*D*). The most highly and significantly elevated LakSol proteins are caspase 1/interleukin 1 beta convertase, apoptosis-associated speck-like protein containing a CARD (PYCARD), cathepsins, and other proteases (Fig. 7*A*), which is consistent with the most prominent pathways in this set. However, the most significantly and highly reduced LakSol proteins are indicative more of iron transport and ion regulation than the KEGG pathways that are numerically most highly represented in the set of reduced LakSol proteins (Fig. 7*B*).

PANTHER enrichment analysis did not yield any significant biological processes or pathways for the sets of elevated and reduced BodHar proteins. However, several KEGG pathways involved in oxidative and cholesterol metabolism and growth are well represented in the set of elevated BodHar proteins whereas cell adhesion, immune-related pathways, apoptosis, necroptosis, and ECM-related KEGG pathways are prominent in the set of reduced BodHar proteins (Fig. 6*E*, 6*F*). The most significantly and highly elevated BodHar proteins represent the above mentioned KEGG pathways of oxidative and lipid metabolism but at least two of these proteins are also indicative of increased demands for long-term osmotic homeostasis in seawater (SW) (glutamine synthetase and antifreeze protein LS12, Fig. 7*C*). Likewise, several proteins that are most highly reduced in BodHar fish, including collagens and proteins involved in cell adhesion and pattern recognition such as transglutaminase 2, clathrins, and alpha-2-macroglobulin represent the corresponding KEGG pathways mentioned above (Fig. 7*D*). Overall, the extent of abundance differences is lower for elevated and reduced BodHar proteins than for other populations (Fig. 4 and 7).

**Fig. 8.**
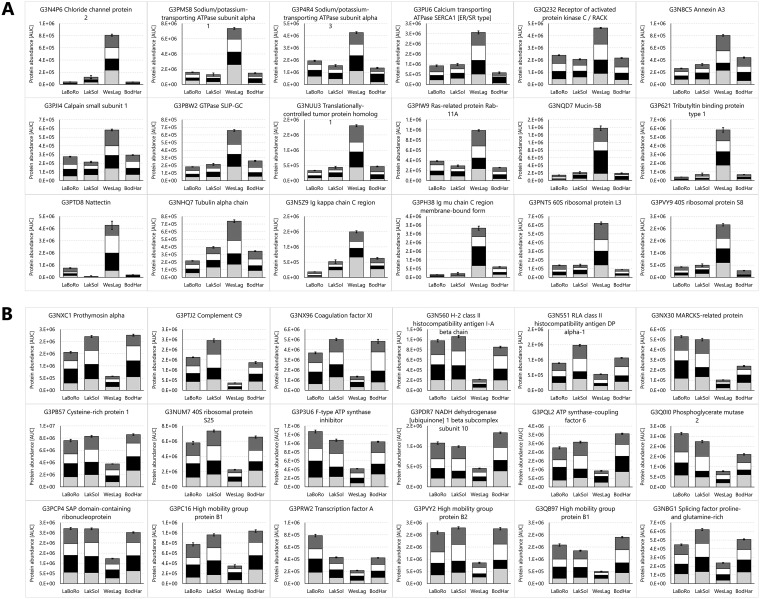
**Proteins from threespine stickleback gills with the greatest and most significant abundance differences in the Westchester Lagoon (WesLag) population compared with the other populations.** Data for significantly elevated (*A*) and reduced (*B*) proteins represent the sum of the means of all four population samplings (PSs). Error bars represent the S.E. of the cumulative dataset from all four PSs (*n* = 24 per population). For each protein the reproducibility of the overall trend can be assessed by inspecting the contributions of individual PSs, which are indicated by different black and white shading intensity (PS1 to PS4 from bottom to top).

PANTHER enrichment analyses for the set of elevated WesLag proteins shows that ribonucleoprotein complex (*p* = 0.0379), hydrolase activity acting on ester bonds (*p* = 0.0385) and phosphatase activity (*p* = 0.027) are enriched whereas integrin signaling (*p* = 0.0341) and transcription (*p* = 0.0404) are depleted (Fig. 5*G*). For the set of reduced WesLag proteins PANTHER analysis indicates enrichment of amino acid metabolism (*p* = 0.0364) and blood coagulation (*p* = 0.00426) but depletion of organelle organization (*p* = 0.0464), transcription factors (*p* = 0.0471) and defense/immunity proteins (*p* = 0.0028) (Fig. 5*H*). KEGG pathways well represented in the set of elevated WesLag proteins include calcium and GTPase signaling pathways, regulation of actin cytoskeleton, focal adhesion, tight junction, ECM receptor interaction, and proteostasis/protein turnover (Fig. 5*G*). Oxidative metabolism, complement and coagulation cascades, RNA transport, endocytosis, adrenergic signaling and phagosome are represented in both elevated and reduced WesLag protein sets, suggesting that these processes are remodeled relative to the other populations. However, there are also KEGG pathways that are only prominent in the set of depleted WesLag proteins including endocannabinoid and PPAR signaling, spliceosome, lysosome, antigen processing and presentation, necroptosis, glutathione metabolism, and pentose phosphate pathway (Fig. 5*H*). KEGG pathways that are prominent in the set of elevated WesLag proteins are exemplified by the most highly and significantly elevated WesLag proteins, including many calcium regulatory proteins and proteins involved in GTP signaling, immunity, cell adhesion, cytoskeletal organization, pattern recognition, and protein translation. In addition, these proteins include ion transporters such as a chloride channel and two subunits of the Na^+^/K^+^-ATPase (Fig. 8*A*). The most highly reduced and significant WesLag proteins mostly reflect the prominent KEGG pathways in this set and include immunity proteins such as complement proteins, thymosin, and MHC proteins, proteins involved in oxidative metabolism, and transcriptional regulators (Fig. 8*B*).

## DISCUSSION

### 

#### 

##### Assay Library Development

DIA targeted data extraction was first introduced six years ago (3). This method eliminates common problems with protein quantitation by DDA (stochastic, irreproducible selection and undersampling of precursor ions) while greatly exceeding the scope (number of proteins) that can be quantified by targeted approaches (SRM, MRM, PRM). Since its inception, many variants of DIA technology have been employed. Two major variants include SWATH on triple-quad instruments and DIA on UHR-qTOF instruments (9, 31–35). Recently, DIA targeted data extraction has been advanced and quantitative assay precision improved by providing tools that permit target list filtering and generation of curated target lists based on stringent quality control criteria, some of which are evaluated using a “training sample set.” In this study we have used Skyline (21) for targeted DIA data extraction and filtering the target list. The resulting assay library is much shorter than the initial raw target list generated from spectral libraries. Quantitative precision of the assay library is greatly improved because protein assignment ambiguities, low-quality and low-signal transitions, and proteins with less than two unique precursors were eliminated during target list filtering. However, target list filtering also eliminates proteins that are potentially biologically interesting and detectable by multiple peptides if not at least two of these peptides meet all filter criteria. Automatic target list filtering with Skyline eliminated most low-quality transitions. Moreover, using all 24 samples from PS1 as a “training sample set” for several automatic steps and a final manual step of target list filtering further eliminated unreliable transitions with ragged peak shapes, interferences, inconsistent peak outlines or low signal-to-noise ratio.

The assay library permits large-scale (network-enabling) analyses of proteome dynamics in gills of threespine sticklebacks exposed to variable ecological contexts. A current limitation is that less than 10% of the overall proteome can be assayed with this assay library. Although it is unlikely that all proteins encoded by the genome are expressed in gills, even greater sensitivity and dynamic range of future instrumentation will gradually alleviate this limitation. The conceptual approach for assay library construction used in our study consisted of first acquiring high-quality fragment ion spectra in DDA mode, second, assigning peptide sequences to these spectra and annotating them to specific proteins, third, generating annotated spectral libraries, and fourth, compiling the final assay from the spectral library. This approach follows a recently described protocol (7, 8). However, here we use PEAKS Studio rather than the transproteomics pipeline (TPP) for consolidating multiple search engine results to annotate MSMS spectra from DDA data and Skyline rather than OpenSWATH (36) for spectral library construction and assay library filtering. Schubert and colleagues filtered out fragments smaller than 350 *m*/*z* or greater than 2000 *m*/*z* as well as precursors that fall within the precursor isolation window from their assay library and they used a mass accuracy threshold within ±0.05 *m*/*z* of the expected mass (8). We used similar criteria for the generation of the initial target list via the transition settings dialogue in Skyline. In step 1 of reducing the initial library to the final assay library the five most intense y and b ions were selected (min. fragment ion = ion 3, *e.g.* y4 even if y2 and y3 are more intense, max. fragment ion = last ion −1 excluding the precursor isolation window, m/z threshold ±0.035 range) (Fig. 1*A*–1*D*). Schubert and colleagues recommend that all assays in the final assay library have the same number of transitions to avoid mixed distributions that complicate statistical analysis. However, our inclusion of some precursors represented by four (rather than the nominal five) transitions in the assay library is accounted for because MSstats employs mixed linear models for statistical analysis (22). Removal of transitions that produce interferences in most or all training samples improves quantitative precision without impairing protein coverage of the assay library. In addition, four processed transitions still provide the highest separation power of the mProphet score (23).

##### Quantitative Reproducibility of the Stickleback Gill Assay Library

The reliability and quantitative reproducibility of the assay library was very high as assessed by analysis of three additional PSs, each consisting of 24 samples that were not included in the “sample training set” derived from PS1. No manual curation of the assay library was performed for any samples in PS2, PS3, and PS4 and all targeted data extraction procedures were done using Skyline automated workflows, including internal retention time alignment, mProphet peak scoring, and MSstats quantitative analysis (21–23). Visual inspection verified that transition peaks were properly assigned and outlined for precursors of proteins that were identified by MSstats analysis as significantly elevated or reduced in a population.

Because all raw data, quantitative results, and metadata are conveniently accessible in Panorama public (37) the corresponding Skyline files can be downloaded directly to reproduce all quantitative and statistical results. Although the population-specific abundance patterns of many proteins were very consistent in all four PSs, there were also differences for many proteins. Most of these differences were small. They can be attributed to three causes: First, individual differences in the expression of at least some proteins are expected between different fish. Second, the level of elevation or reduction of some proteins is slightly different, *i.e.* just above the significance threshold in some and slightly below that threshold in other PSs. Third, different population samplings for each of the four PSs may have pooled different subsets of the population. We view the last possibility as the least likely because all population samplings were performed under very similar conditions for a given population (same temperature, salinity, season, time of day, sampling method).

DIA data sets analyzed with the gill assay library represent a consistent matrix for powerful network modeling approaches, which can be complemented in the future by data sets for additional samples to enable comprehensive modeling of ecological and evolutionary effects on gill proteome dynamics. Although limited to a subset of the proteome (1506 proteins), the gill assay library allows robust and reproducible quantitation of a rich repertoire of striking population-specific proteome signatures. In contrast to genomes that are stable within a single individual, the corresponding proteome is extremely dynamic depending on developmental, ecological, and cell/tissue contexts (38). Therefore, species- and tissue-specific proteomes can be considered excellent “reflections” of an organism's life history exposures and experiences that provide information about its exposome (39, 40). Biological implications of the gill proteome signatures identified in this study are discussed below. An analysis of whether these proteome signatures represent the result of evolutionary/genetic adaptations or physiological plasticity/acclimation exceeds the scope of this study. Nevertheless, the identification of pertinent proteome signatures and the establishment of a gill assay library enables such future studies.

##### Ecological Significance of Population-specific Proteome Signatures

Pathway and GO enrichment approaches represent convenient tools for surveying broad biological implications of large data sets (41). We have used such approaches previously for threespine sticklebacks to interpret large proteomics datasets and condense the associated information (4, 5). However, only known pathways can be analyzed with pathway/GO enrichment approaches and representation of pathways and GO terms in existing databases is highly uneven. Protein annotation with GO terms and pathways is very heterogeneous ranging from none to hundreds per protein. Moreover, functional annotations are often based on only a handful of canonical model organisms studied in particular contexts. Thus, enrichment analyses are biased against poorly annotated pathways/GO terms that may be more novel and unique in the tissue, species, and contexts studied. In addition, because the assay library covers less than 10% of the stickleback proteome the statistical power of enrichment analyses is lower compared with transcriptomics studies that use the entire genome for this purpose. Because of these limitations and caveats of such enrichment analyses we have complemented them with manual, literature-based assessment of pertinent functions of highly significant proteins.

##### Proteome Signature of the Bodega Harbor Population

Marine sticklebacks from Bodega Harbor have the least unique gill proteome of all populations analyzed. A possible reason for this observation is the evolutionarily ancestral state of marine threespine stickleback populations (16), which may include most of the traits displayed by more specialized, derived populations at levels high enough to reflect a “species average.” Despite the lack of significant enrichment/depletion of any PANTHER pathway/GO term, a distinct proteome signature is evident even for this population. Although fewer in number compared with other populations, more than a hundred proteins are significantly elevated and reduced in BodHar fish. Elevated BodHar proteins cluster in KEGG pathways that are indicative of enhanced oxidative and lipid metabolism suggesting that energy use and/or storage in the form of lipids is elevated in the BodHar population. In addition, glutamine synthetase and antifreeze protein L12 promote osmotic homeostasis at elevated salinity by increasing the concentration of compatible osmolytes (42, 43). Reduced BodHar proteins cluster in growth-inhibitory (apoptosis, necroptosis), cell adhesion, extracellular pattern recognition, and ECM-related KEGG pathways. For instance, the marked depletion of transglutaminase 2 indicates reduced needs of the BodHar population for protein cross-linking (44), protection from apoptosis (45), ECM stabilization (46), and tissue damage repair (47). Overall, the BodHar gill proteome signature is consistent with the eco- and morpho-type of the BodHar population and suggests specific molecular targets that might underly enhanced growth and SW adaptation of BodHar fish.

##### Proteome Signature of the LaBoRo Population

The LaBoRo population is unique among threespine sticklebacks because it chronically experiences temperatures of at least 30 °C, which are lethal or highly detrimental for most other stickleback populations (48). Elevated LaBoRo proteins cluster in KEGG pathways that indicate enhanced roles of the extracellular matrix, cell adhesion, the plasma-membrane anchored cytoskeleton, and the complement system. Although many isoforms of common HSPs (HSP70, HSP60, HSP90) are included in the assay library, none of them are significantly elevated in the LaBoRo population. This unexpected finding may indicate that these molecular chaperones are not limiting for proteostasis at chronically elevated temperature. However, two HSP47 (serpin 1) isoforms are strikingly elevated in gills of LaBoRo sticklebacks. HSP47 is a molecular chaperone for collagens and some other ECM proteins that promotes their maturation during thermal stress (49). Although collagens are not among the elevated ECM proteins in LaBoRo fish, many other ECM and cell adhesion proteins are highly elevated suggesting a major role of extracellular matrix stabilization in LaBoRo stickleback gills. A key role of HSP47 for ECM reorganization has also been demonstrated in animal models of human diseases, including cardiac infarction (50), tumorigenesis (51, 52) and renal glomerulonephritis (53). Moreover, HSP47 plays critical roles for skeletal growth, ECM patterning, and fin regeneration in zebrafish (54). Elevated Hsp47 mRNA levels are correlated with increased temperature and heat-shock in many organisms (49, 55, 56), including fish (57), suggesting that the high habitat temperature causes severe ECM strain in gills of LaBoRo sticklebacks.

KEGG pathways that are most prominent among reduced LaBoRo proteins indicate a suppression of proteasome, lysosome, and ribosome functions as well as protein anabolism, adaptive immunity, and necroptosis. In addition, RNA/DNA binding proteins and translation regulators/factors are significantly enriched among reduced LaBoRo proteins supporting the notion of suppressed protein turnover in the LaBoRo population. A striking proteome feature of LaBoRo sticklebacks is the strong depletion of proteins involved in adaptive immunity whereas several complement proteins involved in innate immunity are elevated. Fish are ectotherms and thermal stress effects on the vertebrate complement system have been documented previously (58, 59). It should be pointed out that the fish gill is a highly vascularized tissue and that proteins detected in this study may be localized in branchial epithelial, vascular, and/or extracellular compartments (60). The depletion of adaptive immunity proteins is exemplified by enormously reduced levels of interferon-induced GTP-binding protein Mx. This protein (Mx) is a very potent antiviral defense protein that protects cells from many RNA and DNA viruses (61, 62). Mx interacts with calcium signaling (63) and proteostasis pathways (*e.g.* the ER stress response) (64). Although causality between molecular LaBoRo proteome signatures and temperature is currently unclear, this population chronically experiences temperatures of at least 30 °C, which are lethal or highly detrimental for most other stickleback populations (48). In addition, strong temperature effects on the immune system have been reported for threespine sticklebacks (65) and other fish species (66). Whether caused by high habitat temperature or other environmental factors, the unique signature of immune system proteins in LaBoRo fish suggests that their adaptive immune functions are compromised. Induction of innate immunity via the complement system may represent a compensatory adaptive strategy. Alternatively, activation of the complement system may indicate increased levels of cellular and tissue-damage under the stressful habitat conditions experienced by LaBoRo sticklebacks. Such increased damage would exacerbate the demand for complement clearance of cellular debris (66, 67).

##### Proteome Signature of the WesLag Population

The PANTHER integrin signaling pathway is significantly depleted from the elevated LaBoRo and WesLag proteins. This finding illustrates that this pathway is well represented by the 1506 assay library proteins but not elevated in either population. Significant enrichment of hydrolase and phosphatase activity among elevated WesLag proteins is exemplified by several highly elevated ATPases that are central for ion transport. The ribonucleoprotein complex is also enriched among elevated WesLag proteins, which cluster into calcium and GTPase signaling, actin cytoskeleton regulation, cell adhesion, pattern recognition, and protein translation KEGG pathways. The most highly elevated WesLag protein is chloride channel protein 2 (CLCN2). This ion channel and the elevated cation ATPases are regulated by environmental salinity in euryhaline fish, including sticklebacks (13, 60, 65, 68). We interpret these findings as evidence that WesLag fish experience frequent salinity fluctuations in their habitat, either because of tidal action or because of migrations between freshwater, brackish, and marine habitats. This anadromous population may have migrated just prior to collection from seawater to Westchester Lagoon to breed. Intracellular calcium, which controls many physiological processes of euryhaline fish including osmoregulation, is also regulated by the Ca^2+^-ATPase (69). Besides calcium signaling, a prominent signal transduction feature of the WesLag-specific gill proteome signature are various GTPases, including several Ras-related small GTPases that are known to regulate structural stability of the cytoskeleton and epithelial homeostasis and barrier function (70–72). These processes are key targets during cellular osmoregulation (73).

PANTHER enrichment of amino acid metabolism and blood coagulation among reduced WesLag proteins is accompanied by clustering of these proteins within endocannabinoid and PPAR signaling, spliceosome, lysosome, antigen processing and presentation, necroptosis, glutathione metabolism, and pentose phosphate KEGG pathways. Suppression of these processes in the WesLag population is supported by the functions of most significantly reduced proteins, for instance the marked depletion of proteins involved in immunity or RNA splicing. Reduced RNA processing and lower levels of complement and MHC proteins in this population indicate a lower demand for scavenging functions such as cleaning up and compensating cellular damage and pathogen debris by the complement system (67). Such functions may be more pronounced in the other populations if they experience greater levels of environmental stress and are more susceptible to pathogen invasion and cellular damage. Interestingly, the regulation of complement proteins and immunoglobulins is opposite in the WesLag population compared with the LaBoRo population, *i.e.* WesLag immunoglobulins are elevated whereas complement proteins are reduced. Whether temperature, specific pathogens, or other environmental factors are the cause for this proteome pattern remains to be determined.

##### Proteome Signature of the LakSol Population

Elevated LakSol proteins are enriched for PANTHER hydrolase and peptidase activities and cluster predominantly in the KEGG pathways ribosome, lysosome, phagosome, antigen processing/presentation, apoptosis and necroptosis. The most highly and significantly elevated LakSol proteins are caspase 1/interleukin 1 beta convertase, apoptosis-associated speck-like protein containing a CARD (PYCARD), cathepsins, and other proteases. The inflammasome comprises a multimeric protein complex consisting of caspase 1, PYCARD, and a nod-like receptor protein (NLRP, *e.g.* NLRP3) (74). Although not present in the assay library, the NLRP sensor of LakSol stickleback gill inflammasomes is likely NLRP3 because this sensor preferentially interacts with the PYCARD adaptor (75, 76). The PYCARD adaptor activates the effector caspase 1 leading to proteolytic cleavage of an inactive cytokine precursor into active, pro-inflammatory interleukin-1 beta (74, 77–79). Inflammasome induction represents a response to parasitism in many species, including fishes (80–82). Freshwater sticklebacks are frequently parasitized (83) and we did observe cestode parasites (*Schistocephalus solidus*) in the body cavity of some LakSol sticklebacks during dissection, but never in any of the other populations. We excluded heavily parasitized fish during dissection but defense mechanisms against parasitism may be even more effective in fish from the same habitat that lack visible signs of parasitism. Potent inflammasome induction has also been reported in response to mercury exposure (84, 85). This finding is significant because Lake Solano is characterized by high mercury concentration resulting from historical gold mining activities in California (86). Intriguingly, cathepsins, which are also elevated in gills of LakSol sticklebacks, regulate the appearance and severity of mercury-induced inflammation (84). In addition, cathepsins are known to participate in inflammasome pathways in many other contexts (87–89).

Reduced LakSol proteins are depleted in PANTHER nucleic acid and RNA binding proteins suggesting that the levels of these proteins are maintained like those in other populations. Instead, reduced LakSol proteins cluster in focal adhesion, ECM-receptor interaction, tight junction, PI3K-Akt signaling, actin cytoskeleton regulation, and oxidative metabolism KEGG pathways. A reduction of oxidative metabolism is supported by a decrease in hemoglobin isoforms. Decreases in hemoglobin and erythrocytes have been observed because of parasitism in freshwater fish (90). For instance, the Brazilian cultivated sport fish *Leporinus microcephalus* has a reduced hematocrit, erythrocyte count, and hemoglobin concentration caused by the parasite *Goezia leporini* (91). Depletion of hemoglobins, iron transporting ferritins, and other plasma proteins may also be a consequence of parasitism in LakSol sticklebacks, but this hypothesis remains to be tested. As expected, proteins responsible for active NaCl secretion (*e.g.* Na^+^/K^+^/2Cl^−^ cotransporter) are reduced in LakSol fish indicating that their gills operate in ion absorption mode (60, 92) in their FW habitat.

##### Conclusions and Perspective

To enable reliable and reproducible quantitation of a consistent set of proteins based on identical criteria in every sample we have developed an assay library for threespine stickleback gills. This resource empowers systematic large-scale analyses of the ecological context-dependence of gill proteome dynamics in threespine sticklebacks. The use of this assay for ecological proteomics was demonstrated by comparing gill proteomes of four populations of threespine sticklebacks. Discrete and ecophysiologically meaningful proteome signatures of locally adapted populations have been identified. These signatures allow deep insight into mechanisms of biochemical adaptation and physiological plasticity in different environments. Future use of this assay library permits reliable and comparable assessment of the effects of any biological context on the gill proteome of threespine sticklebacks. For instance, it can be used in conjunction with controlled acclimation experiments that permit isolation of specific environmental variables such as salinity, temperature, and parasitism to correlate them with gill proteome signatures. Moreover, the gill assay represents a model for construction of assay libraries for other tissues. Multi-tissue assays can be used jointly to capture highly complex, ecologically relevant features of whole organism proteome dynamics. Moreover, a multi-tissue strategy combined with network modeling approaches will reveal environmental impacts on holistic scale proteome dynamics. Such standardized systems-level analyses promise to provide extensive insight into ecological and evolutionary impacts on complex vertebrates.

## DATA AVAILABILITY

Raw mass spectrometry data, metadata, and processed data are available at the following public repositories: MassIVE, ID MSV000081795 (ftp://massive.ucsd.edu/MSV000081795); ProteomeXchange, ID PXD008395 (http://proteomecentral.proteomexchange.org/cgi/GetDataset?ID=PXD008395); CAMP proteome, AC CAMPDDA00023 (https://kueltzlab.ucdavis.edu/CAMP_dda_profiles.cfm?AC=CAMPDDA00023); Panorama Public (https://panoramaweb.org/labkey/JoLi-01.url); CAMP proteome, AC CAMPDIA00001 (https://kueltzlab.ucdavis.edu/CAMP_dia_exp.cfm?ac=CAMPDIA00001).

## Supplementary Material

supplemental Fig. S1
