# Supplementary material for: Development of a Gill Assay Library for Ecological Proteomics of Threespine Sticklebacks (Gasterosteus aculeatus)
Source: Mol Cell Proteomics. 2018 Aug 9;17(11):2146–63. doi: 10.1074/mcp.RA118.000973 (PMC6210217; doi:10.1074/mcp.RA118.000973)
Supplement: supplemental Fig. S1 [file RA118.000973_index.html]

Supplement to Development of a gill assay library for ecological proteomics of threespine sticklebacks (Gasterosteus aculeatus) | Molecular & Cellular Proteomics

## Supplemental Data

- Supplemental Figure 1 - Sampling locations and habitat conditions for the 4 populations of three-spined sticklebacks analyzed in this study.
- Supplemental Figure 2 - Phenotypic traits for the 4 populations of three-spined sticklebacks analyzed in this study.
- Supplemental Table 1 - PEAKSQ Top3 MS1 semiquantitative profiling data
- Supplemental Table 3 - KEGG (Kyoto Encyclopedia of Genes and Genomes) orthology (KO) and PANTHER (Protein Annotation Through Evolutionary Relationships) identifiers for the 1506 assay library proteins
- Supplemental Table 2 - Scaffold peptide ID, protein ID, and semiquantitative spectral counting data
- Supplemental Table S4 - Skyline generated fold changes (FC) and multiple testing corrected significance values of elevated (FC &gt; 2.0) and reduced (FC &lt; 0.5) proteins for all populations and population samplings.
- Supplemental Table S5 - Skyline generated raw data for the cumulative quantitative analysis of all population samplings. Data for all significantly elevated and reduced proteins are shown.
