# Supplementary material for: Development of a Gill Assay Library for Ecological Proteomics of Threespine Sticklebacks (Gasterosteus aculeatus)
Source: Mol Cell Proteomics. 2018 Aug 9;17(11):2146–63. doi: 10.1074/mcp.RA118.000973 (PMC6210217; doi:10.1074/mcp.RA118.000973)
Supplement: supplemental Fig. S1 [file 139476_1_supp_181265_pd41z7.pdf]

Bodega Harbor (**BodHar**)

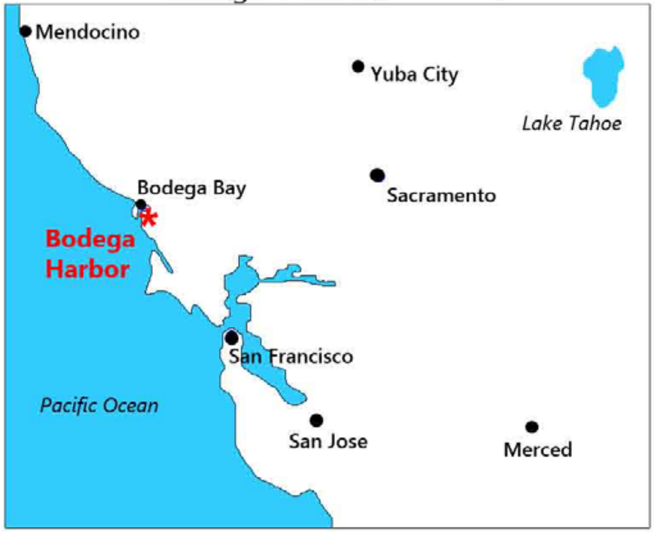

Date collected:
June 18th, 2014

Salinity:
32-34 g/kg

Annual mean temperature:
10-12°C

Temperature at collection:
12°C

Latitude:
38.33°N

Morphotype:
Fully plated

Ecotype:
Resident marine

Lake Solano (**LakSol**)

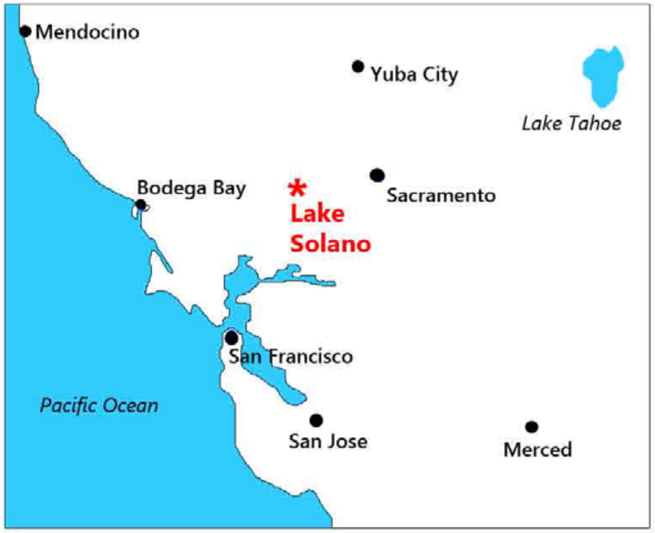

Date collected:
June 25th, 2014

Salinity:
<0.1g/kg

Annual mean temperature:
11-14.72°C

Temperature at collection:
14.5°C

Latitude:
38.49°N

Morphotype:
Low plated

Ecotype:
Resident freshwater

Laguna de la Bocana del Rosario (**LaBoRo**)

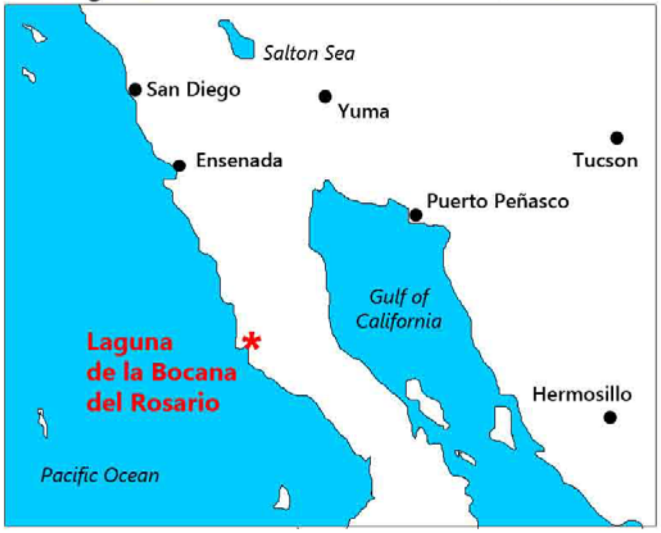

Date collected:
August 30th, 2014

Salinity:
3.4-10.7g/kg

Annual mean temperature:
25-30°C

Temperature at collection:
28.9-29.8°C

Latitude:
30.02°N

Morphotype:
Low plated

Ecotype:
Brackish water

Westchester Lagoon (**WesLag**)

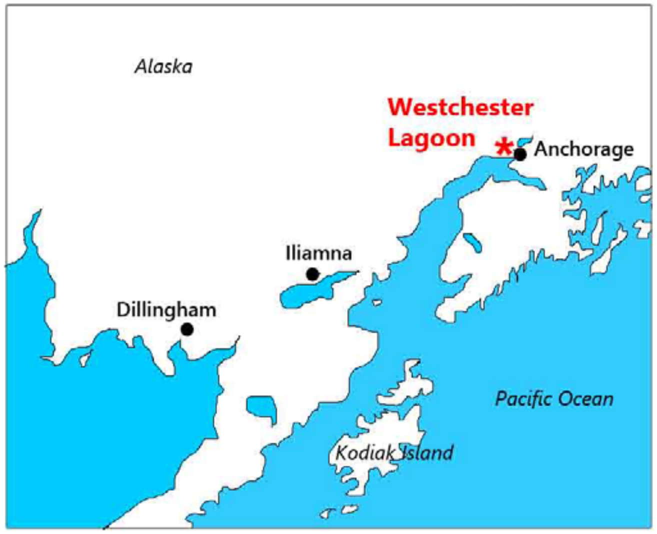

Date collected:
June 7th, 2015

Salinity:
1.4-3.7g/kg

Annual mean temperature:
-.55-15.5°C

Temperature at collection:
15.2°C

Latitude:
61.22°N

Morphotype:
Fully plated

Ecotype:
Brackish water
