# Supplementary figures and images for: Development of a Gill Assay Library for Ecological Proteomics of Threespine Sticklebacks (Gasterosteus aculeatus)
Source: Mol Cell Proteomics. 2018 Aug 9;17(11):2146–63. doi: 10.1074/mcp.RA118.000973 (PMC6210217; doi:10.1074/mcp.RA118.000973)

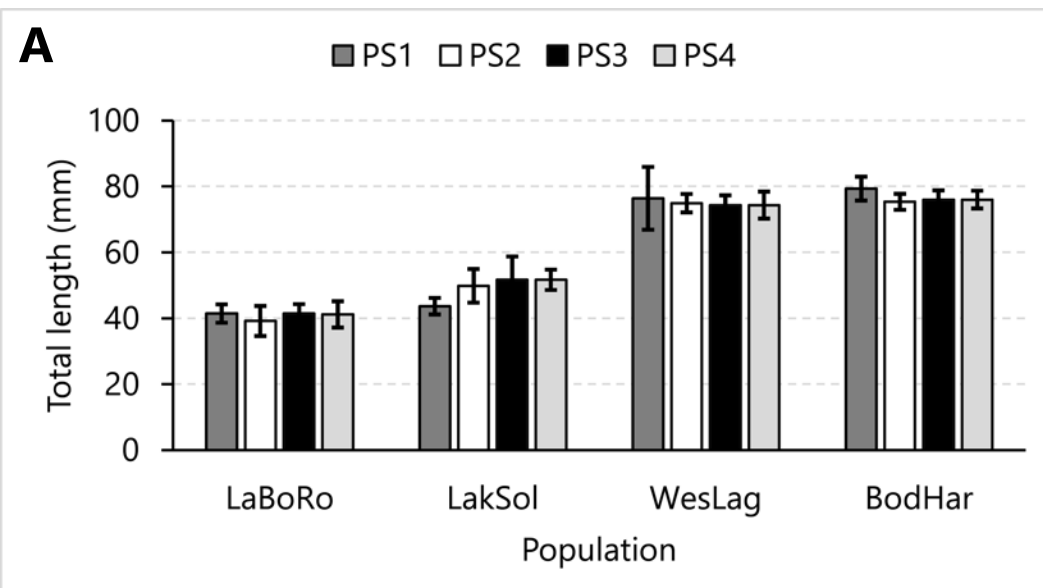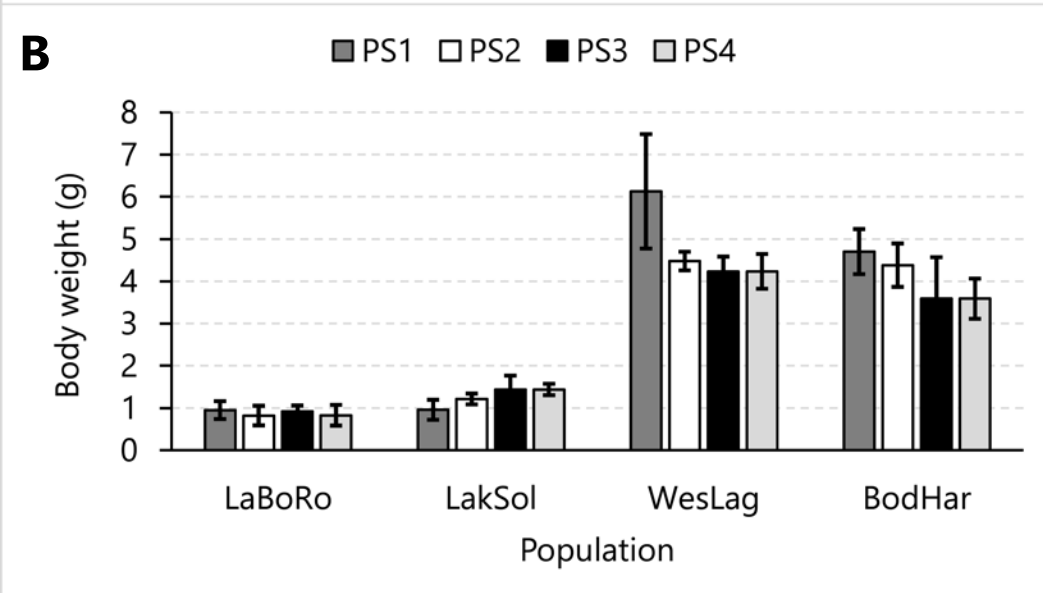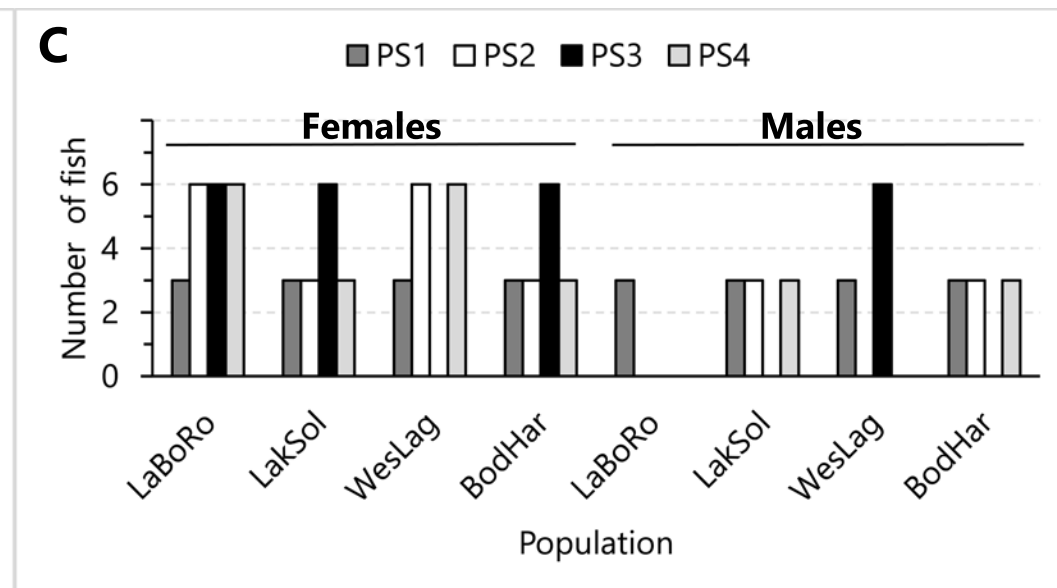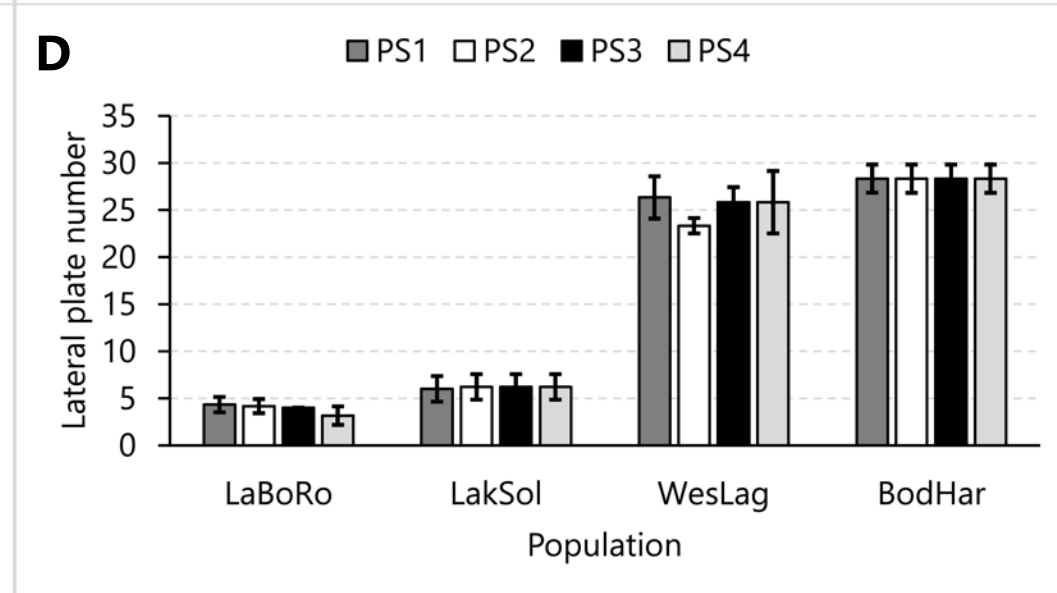

Supplement: supplemental Fig. S1 [file 139476_1_supp_181266_pd41z7.pdf]
